# Estimating genetic diversity and population structure of 22 chicken breeds in Asia using microsatellite markers

**DOI:** 10.5713/ajas.19.0958

**Published:** 2020-04-13

**Authors:** Hee-Jong Roh, Seung-Chang Kim, Chang-Yeon Cho, Jinwook Lee, Dayeon Jeon, Dong-kyo Kim, Kwan-Woo Kim, Fahmida Afrin, Yeoung-Gyu Ko, Jun-Heon Lee, Solongo Batsaikhan, Triana Susanti, Sergey Hegay, Siton Kongvongxay, Neena Amatya Gorkhali, Lan Anh Nguyen Thi, Trinh Thi Thu Thao, Lakmalie Manikku

**Affiliations:** 1 Animal Genetic Resources Center, National Institute of Animal Science, RDA, Hamyang 50000, Korea; 2 Division of Animal and Dairy Science, Chungnam National University, Daejeon 34134, Korea; 3 Production and technology, National Centre for Livestock Genebank, Ulaanbaatar, 210349, Mongolia; 4 Indonesia Research Institute for Animal Production, Bogor, 16720, Indonesia; 5 Institute of Biochemistry & Physiology, National Academy of Science of Kyrgyzstan, Bishkek, 720071, Kyrgyzstan; 6 Livestock Research Centre, Vientiane, 7170, Lao People’s Democratic Republic; 7 Animal Breeding Division, Nepal Agricultural Research Council, Kathmandu, 44600, Nepal; 8 Department of Animal Breeding and Genetics, Institute of Animal Sciences for Southern Vietnam, Binh Duong 75000, Vietnam; 9 Department of Animal Production and Health, Veterinary Research Institute, Colombo, 20400, Sri Lanka

**Keywords:** Asian Chicken Breeds, Genetic Diversity, Genetic Relationship, Microsatellite Markers, Heterozygosity, Polymorphism Information Content

## Abstract

**Objective:**

Estimating the genetic diversity and structures, both within and among chicken breeds, is critical for the identification and conservation of valuable genetic resources. In chickens, microsatellite (MS) marker polymorphisms have previously been widely used to evaluate these distinctions. Our objective was to analyze the genetic diversity and relationships among 22 chicken breeds in Asia based on allelic frequencies.

**Methods:**

We used 469 genomic DNA samples from 22 chicken breeds from eight Asian countries (South Korea, KNG, KNB, KNR, KNW, KNY, KNO; Laos, LYO, LCH, LBB, LOU; Indonesia, INK, INS, ING; Vietnam, VTN, VNH; Mongolia, MGN; Kyrgyzstan, KGPS; Nepal, NPS; Sri Lanka, SBC) and three imported breeds (RIR, Rhode Island Red; WLG, White Leghorn; CON, Cornish). Their genetic diversity and phylogenetic relationships were analyzed using 20 MS markers.

**Results:**

In total, 193 alleles were observed across all 20 MS markers, and the number of alleles ranged from 3 (MCW0103) to 20 (LEI0192) with a mean of 9.7 overall. The NPS breed had the highest expected heterozygosity (*H*_exp_, 0.718±0.027) and polymorphism information content (PIC, 0.663±0.030). Additionally, the observed heterozygosity (*H*_obs_) was highest in LCH (0.690±0.039), whereas WLG showed the lowest *H*_exp_ (0.372±0.055), *H*_obs_ (0.384±0.019), and PIC (0.325±0.049). Nei’s DA genetic distance was the closest between VTN and VNH (0.086), and farthest between KNG and MGN (0.503). Principal coordinate analysis showed similar results to the phylogenetic analysis, and three axes explained 56.2% of the variance (axis 1, 19.17%; 2, 18.92%; 3, 18.11%). STRUCTURE analysis revealed that the 22 chicken breeds should be divided into 20 clusters, based on the highest ΔK value (46.92).

**Conclusion:**

This study provides a basis for future genetic variation studies and the development of conservation strategies for 22 chicken breeds in Asia.

## INTRODUCTION

Prior to the Convention on Biological Diversity (CBD; Rio de Janeiro, Brazil) in 1992, genetic resources had been recognized as common global resources, however, after the CBD, they began to be regarded not as common, but as the individual resources of each respective country. In 2007, the Interlaken Declaration was adopted by the United Nations Food and Agriculture Organization, which suggested that each country should preserve their own animal genetic resources and promote the sustainable use of local breeds [1]. Moreover, the Nagoya protocol on access to genetic resources and the fair and equitable sharing of their benefits, for the conservation and sustainable use of biodiversity [2], was adopted in October 2010 by the CBD, at the 10th Conference of the Parties. The importance of animal genetic resources has subsequently become established.

Animal genetic diversity is a source of raw genetic material that can be utilized to improve breeds and adapt livestock populations to changing environments and demands. Thus, acquiring information on animal genetic diversity is essential to design strategies for their sustainable management [3,4]. In Asia, there are many chicken breeds that are distinguished by phenotypic differences, such as feather color, shank color, and comb type. While there are more than 21 billion chickens in the world, more than half of these (53%) are found in Asia. Among the common types of livestock kept by humans, chickens have the largest number of different breeds, at approximately 1,669, of which 1,514 are local breeds and 155 are regional or from areas that cross international boundaries [5]. However, due to the spread of imported breeds that have good commercial performance, the local breeds with poorer commercial performance have been ignored, to the point that some are now threatened by extinction [6]. The loss of a breed to extinction means the loss of its unique genetic resources, such as environmental adaptability and resistance to endemic diseases [7]. Therefore, it is necessary to develop a conservation strategy for local breeds, by studying their genetic diversity.

To identify genetic uniqueness, many countries have evaluated the genetic diversity and relatedness of local breeds using DNA markers such as microsatellites (MS), mitochondrial DNA (mtDNA), copy number variation, and single nucleotide polymorphism (SNP) [8–11]. In recent years, SNPs have been widely used in genetic research. MS markers are comparatively cheap to genotype and provide more genetic information for the population per marker than SNPs, which are biallelic markers. Moreover, MS markers are easily typed in samples with low concentrations of DNA and enable quick identification of breeds in contrast to SNPs [12]. MS markers, also known as simple-sequence repeats, have short tandem repeats of approximately 2 to 6 bp, and because they show co-dominant inheritance, are highly polymorphic, and are distributed throughout the genome [13,14], they are widely used to assess genetic diversity and relationships in many different fields [15–18].

Although many studies have analyzed the genetic diversity and phylogenetic relatedness of chickens using MS markers, they have been limited, as their samples generally only come from breeds of their respective country or of a few countries [19–22]. The National Institute of Animal Science (NIAS) in South Korea, however, has been carrying out the Asian Food & Agriculture Cooperation Initiative (AFACI) Animal Genetic Resources (AnGR) project since 2016, for the purpose of improving the value of animal genetic resources across Asia. Currently, 12 countries, including South Korea, are designated as member countries; NIAS has established a cooperative system by providing information and technologies for the characterization of animal genetic resources from these countries. NIAS is consequently able to utilize a large number of Asian chicken breed samples for scientific research. Consequently, in this study, we have investigated the genetic diversity and relationships among 22 chicken breeds in eight AFACI member countries including three imported breeds, using 20 MS markers.

## MATERIALS AND METHODS

### Sample collection and extraction of genomic DNA

A total of 469 chickens, belonging to 19 different chicken breeds from eight different countries and three imported breeds were used in this study. The 22 chicken breeds included six Korean native breeds (KNG, KNB, KNR, KNW, KNY, KNO), four Laotian (LYO, LCH, LBB, LOU), three Indonesian (INK, INS, ING), two Vietnamese (VTN, VNH), one Kyrgyzstani (KGPS), one Mongolian (MGN), one Nepalese (NPS), one Sri Lankan (SBC), and three imported (RIR, Rhode Island Red; WLG, White Leghorn; CON, Cornish). Detailed information on these breeds can be found in Table 1. Ulnar venous blood of the six Korean native breeds and the three imported breeds was collected from the Animal Genetic Resources Research Center at NIAS. Genomic DNA was extracted from the blood using the Wizard Genomic DNA purification Kit (Promega, Madison, WI, USA) according to the manufacturer’s instructions. Genomic DNA of the other chicken breeds was obtained from each country, for the purposes of the AFACI AnGR project. The DNA concentrations were quantified by UV Spectrophotometer (Nanodrop ND-1000; Thermo Scientific, Waltham, MA, USA) and the samples were diluted to a final concentration of 10 ng/μL in distilled water. This experiment was conducted with the approval of the NIAS Committee on the Ethics of Animal Experiments (approval number: 2018-048).

### Microsatellite markers and polymerase chain reaction amplification

Ten MS markers were selected from among the International Society for Animal Genetics / Food and Agriculture Organization of the United Nations (ISAG/FAO) recommended markers. Another Ten MS markers were selected based on their high heterozygosity in the Ark database website (Roslin Bioinformatics Group, Edinburgh, UK). The information for the twenty MS markers used in this study is available in Supplementary Table A. Extracted DNAs were amplified by the GeneAmp PCR 9700 system (Applied Biosystems, Foster, CA, USA) using AccuPower Negative dye PCR PreMix (Bioneer, Daejeon, Korea), including DNA polymerase, dNTP, Tris-HCl, KCL, and MgCl_2_. The polymerase chain reaction (PCR) reactions were performed in a total reaction volume of 20 μL containing 2 μL of template DNA, and 0.4 to 2.6 μL (2 pmol/μL) of primer based on the multiplex combinations. The initial denaturation was performed at 95°C for 5 min, followed by 35 cycles of 60 s at 95°C, 45 s of annealing at 58°C to 62°C based on the multiplex combination, 60 s of extension at 72°C, a final extension at 72°C for 30 min, and then cooling to 4°C.

### Determining allele sizes in each marker

After PCR amplification, the genotyping reaction mixtures were made using 1 μL of the PCR products, 10 μL of Hi-Di Formamide (Applied Biosystems, USA), and the GeneScan 500 LIZ Size Standard (Applied Biosystems, USA) mixture. The genotyping reaction mixture was denatured for 10 min at 95°C and then immediately placed in ice. Electrophoresis was performed using capillary arrays in an ABI PRISM 3130xl Genetic Analyzer (Applied Biosystems, USA). The allele sizes were determined using GeneMapper Software 5 (Applied Biosystems, USA) and was analyzed statistically.

### Statistical analysis

The allele frequencies, the number of alleles, expected heterozygosity (*H*_exp_), observed heterozygosity (*H*_obs_), and polymorphism information content (PIC) values for each of the chicken breeds across the 20 loci were calculated using the MS Tool Kit [23]. Nei’s D_A_ genetic distances between breeds were calculated using the DISPAN software [24]. The output file for the neighbor-joining (NJ) phylogenetic tree was generated using the PHYLIP package [25] and visualized using TreeView 1.6 [26].

The genetic structures and the degree of admixture among the 22 chicken breeds were analyzed using the Bayesian clustering procedure of STRUCTURE ver 2.3.4 [27]. Twenty independent runs were performed for each K value from 2 to 22. For all runs, the admixture models had a burn-in period of 20,000 repeats, followed by 100,000 repeats of the Markov chain Monte Carlo algorithm. To identify the K value that best fits the data, STURCTURE HARVERSTER [28] was used, which implements the Evanno method [29]. The CLUMPP program ver 1.1.2 [30] was used to align the 20 repetitions of each K value. The CLUMPP output files were visualized using the DISTRUCT program ver 1.1 [31]. Principal coordinate analysis (PCoA) was conducted using the adegenet package [32] in R Studio [33].

## RESULTS AND DISCUSSION

### Genetic diversity of 22 chicken breeds using MS markers

To obtain insight into the genetic diversity and population structures, the *H*_exp_, *H*_obs_, and the PIC value for each locus were calculated using the MS Tool kit (Table 2). From the 20 MS markers, a total of 193 alleles were identified in the 22 chicken breeds. The number of alleles ranged from 3 (MCW0103) to 20 (LEI0192) (mean 9.65). The means of *H*_exp_, *H*_obs_, and PIC were 0.598±0.022, 0.591±0.020, and 0.523±0.023, respectively, for the 20 MS markers. The lowest values of *H*_exp_ (0.348±0.036), *H*_obs_ (0.382±0.046), and PIC (0.272±0.027), were all found at the MCW0103 locus, whereas the highest values of *H*_exp_ (0.714±0.021) and PIC (0.640±0.022) were found at the ADL0176 locus, and the highest value of *H*_obs_ (0.735±0.034) was found at the MCW0193 locus. Moreover, MCW0103 showed low polymorphism levels in previous studies [34–36]. Botstein et al [37] reported that MS markers with PIC≥0.5 and *H*_exp_≥0.6 were highly informative for genetic analysis. Our study demonstrated that 13 of the 20 MS markers were highly informative for discrimination analysis, and would be appropriate for the analysis of the 22 chicken breeds.

Furthermore, the genetic diversity parameters of the 22 chicken breeds were calculated using the MS Tool Kit (Table 3). The mean number of alleles ranged from 2.15±0.93 (MGN) to 6.50±3.02 (NPS). The imported breed WLG showed the lowest *H*_exp_ (0.372±0.055), *H*_obs_ (0.384±0.019) and MGN showed the lowest PIC (0.306±0.048), while NPS showed the highest *H*_exp_ (0.718±0.027) and PIC (0.663±0.030). Correspondingly, although the population size was small, LCH had the highest *H*_obs_ (0.690±0.039). Overall, the diversity of the imported breeds was lower than that of local chicken breeds in 8 countries, except for MGN. This is because the imported breeds have been strongly selected for their performance characteristics and breeding purpose (meat type, egg type, etc.) for many decades. In our study, the genetic diversity of the Korean native chicken (KNC) breeds was lower than that of the other Asian chicken breeds tested. The reason for this may be that five KNC breeds (KNY, KNR, KNW, KNG, and KNG) were restored in 2008 by NIAS, according to the genetic fixation of their different feather colors [38], and this fixation was absent in the other Asian chicken breeds tested. Thus, we further investigated whether the genetic distances could be discriminated amongst between the 22 chicken breeds.

### Genetic distance and phylogenetic analysis among 22 chicken breeds

**Figure 1:**
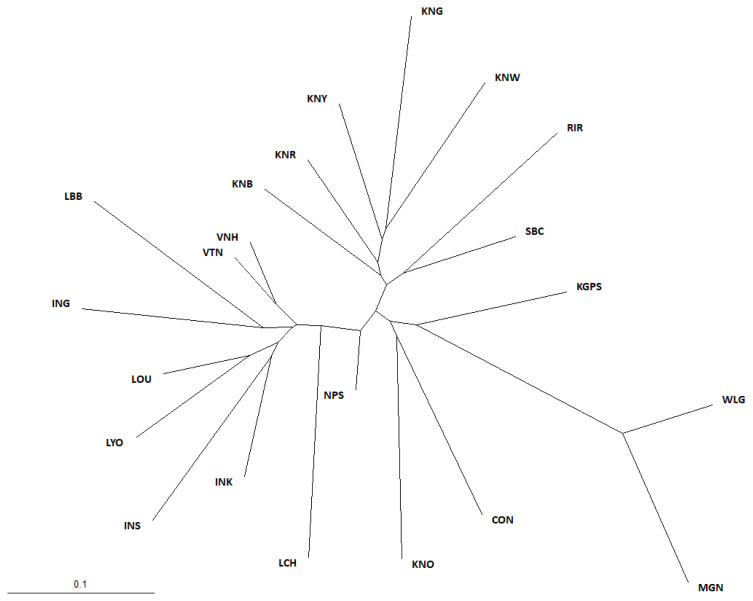
Neighbor-joining phylogenetic tree showing genetic relationships among 22 chicken breeds based on D_A_ genetic distance. It showing the genetic relationships among 22 chicken breeds. RIR, Rhode Island Red; WLG, White Leghorn; CON, Cornish; KNG, Korean Grayish-brown; KNB, Korean Black; KNR, Korean Reddish-brown; KNW, Korean White; KNY, Korean Yellowish-brown; KNO, Korean Ogye; MGN, Mongolian Nuthiin bor; INK, Indonesian KUB; INS, Indonesian Sensi; ING, Indonesian Gaok; KGPS, Kyrzyzstani GPS-H; LYO, Laotian York; LCH, Lotian Chae; LBB, Laotian Black Bone; LOU, Latotian Ou; NPS, Nepalese Sakini; SBC, Sri Lankan Junglefowl.

To further investigate the genetic divergences among the breeds using the 20 MS marker allele frequencies, we estimated Nei’s D_A_ genetic distance between pairs of breeds, for all 22 chicken breeds, using the DISPAN program (Supplementary Table B); the shortest genetic distance was between VNH and VTN at 0.086, and the longest was between KNG and MGN at 0.503. Moreover, to understand the evolutionary relationships among the chicken breeds, an NJ phylogenetic tree was constructed using the PHYLIP program based on the D_A_ genetic distance (Figure 1). In our study, three main branches appear in the phylogenetic tree. The first main branch comprised the KNC breeds (except for KNO), and the RIR and SBC breeds. The WLG, CON, KNO, MGN, and KGPS breeds constituted the second major branch, and this branch was further subdivided into the WLG and CON groups. KNO was grouped with CON, whereas MGN and KGPS were grouped with WLG. The third main branch was comprised of the other Asian chicken breeds. This suggests that the KNC breeds are clearly genetically separated from the other Asian chicken breeds that we studied.

### Clustering and principal coordinate analysis

**Figure 2:**
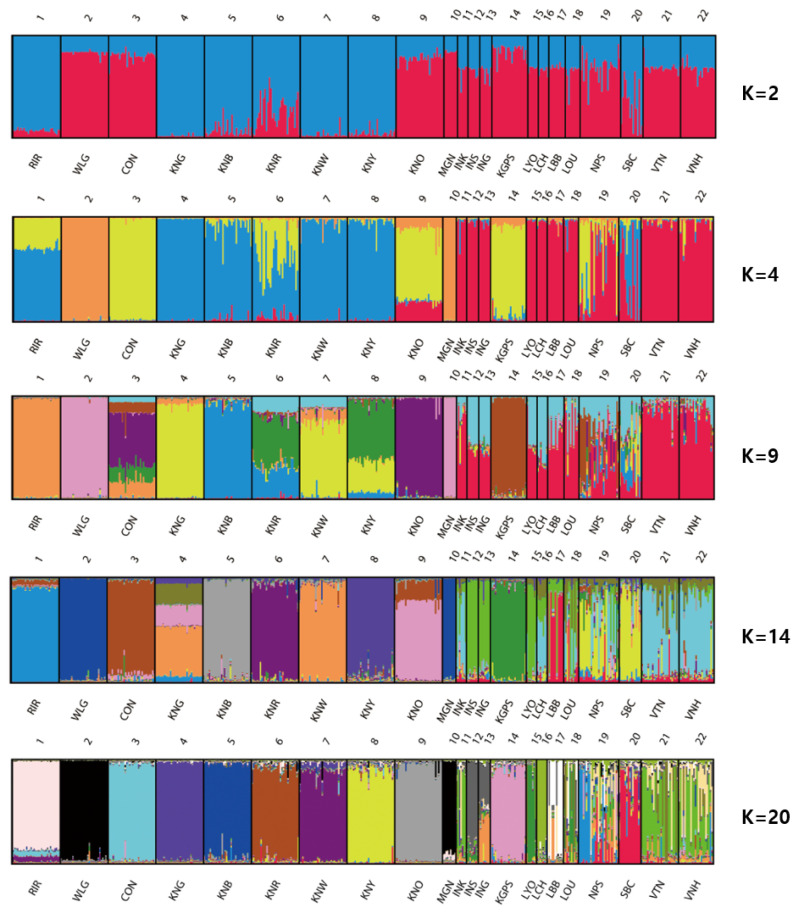
Clustering assignment of the 22 chicken breeds obtained by STRUCTURE analysis. Each of the 469 chickens is represented by a thin vertical line, which is divided into colored segments which represent the proportional contribution of the inferred K = 4, 9, 14, 20 clusters. The populations are separated by thin vertical black lines. RIR, Rhode Island Red; WLG, White Leghorn; CON, Cornish; KNG, Korean Grayish-brown; KNB, Korean Black; KNR, Korean Reddish-brown; KNW, Korean White; KNY, Korean Yellowish-brown; KNO, Korean Ogye; MGN, Mongolian Nuthiin bor; INK, Indonesian KUB; INS, Indonesian Sensi; ING, Indonesian Gaok; KGPS, Kyrzyzstani GPS-H; LYO, Laotian York; LCH, Lotian Chae; LBB, Laotian Black Bone; LOU, Latotian Ou; NPS, Nepalese Sakini; SBC, Sri Lankan Junglefaowl.

We conducted clustering analysis using Bayesian clustering, which provided more accurate estimates of relatedness of the breeds [39]. According to the STRUCTURE analysis the most probable number of inferred clusters and the K value (ΔK), was K = 20 (46.92). The genetic structures of each chicken breed (for K = 2, 4, 9, 14, and 20) were visualized using DISTRUCT (Figure 2). For K = 2, the KNC breeds (excepting KNO) and SBC were clustered with RIR, whereas the other Asian chicken breeds were clustered with WLG and CON. WLG and MGN were distinguished from the other breeds at K = 4. These two breeds were found to differ the most in terms of genetic composition, compared with the other breeds. Differentiation from the other breeds began for RIR and KGPS at K = 9, and for CON at K = 14. Based on the CLUMPP analysis at K = 20 (Supplementary Table C), 11 breeds (RIR, WLG, CON, KNG, KNB, KNR, KNW, KNY, KNO, KPGS, and LCH) were detected in independent cluster and each of these breeds occurred predominantly in one cluster (with more than 84% of its membership in one cluster). Moreover, MGN had 88.9% and WLG had 95.1% membership in cluster 18, and the genetic distances between them were short (0.193). It was difficult to distinguish between WLG and MGN, suggesting that MGN was derived from WLG. SBC was also detected independently in cluster 1, but with a relatively low proportion of membership (68.3%). The proportion of membership of the other breeds ranged from 25.3% to 79.5% in cluster 13 (LOU) and 17 (INS), respectively. Generally, the genetic uniformity of the imported breeds (RIR, WLG, CON) and KNC breeds was higher than that of the other Asian chicken breeds, except for MGN, KPGS, and LCH. This is probably because these Asian chicken breeds have not gone through genetic fixation processes via strong selection processes. It means they were crossbreed. If genetic uniformity is low, it is difficult to determine whether a breed is distinct from other breeds. Therefore, to increase genetic uniformity, as long as it does not reduce genetic diversity through planned breeding, these Asian chicken breeds should be selected according to their specific purposes.

**Figure 3:**
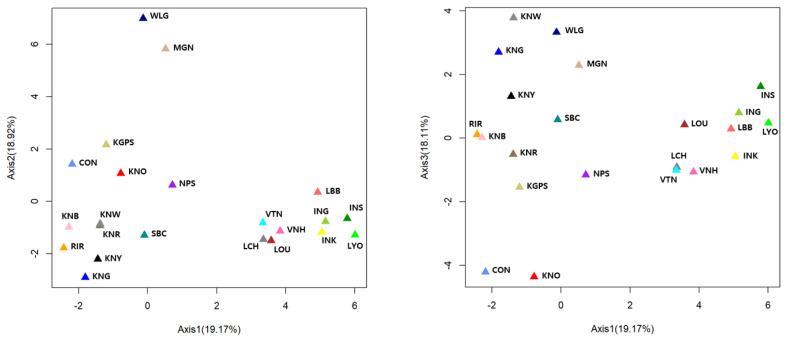
Principal coordinate analysis (PCoA) of allele frequencies from 20 microsatellite markers in 22 chicken breeds. The percentage of the variation explained by the plotted principal coordinates is indicated on the axes. RIR, Rhode Island Red; WLG, White Leghorn; CON, Cornish; KNG, Korean Grayish-brown; KNB, Korean Black; KNR, Korean Reddish-brown; KNW, Korean White; KNY, Korean Yellowish-brown; KNO, Korean Ogye; MGN, Mrtmiongolian Nuthiin bor; INK, Indonesian KUB; INS, Indonesian Sensi; ING, Indonesian Gaok; KGPS, Kyrzyzstani GPS-H; LYO, Laotian York; LCH, Lotian Chae; LBB, Laotian Black Bone; LOU, Latotian Ou; NPS, Nepalese Sakini; SBC, Sri Lankan Junglefowl; VTN, Vietnamese Ninh Hoa; VNH, Vietnamese Ninh Hoa.

To assess the relatedness of breeds, we carried out PCoA analysis using the allele frequencies of the 20 MS markers (Figure 3). These results were similar to those of our phylogenetic tree and structure analysis. The percentages in the label of each axis indicate the variance explained by the axis. Three axes explained 56.2% of the variance; the first two explained 19.17% and 18.92%, respectively, and the third axis explained 18.11%. Remarkably, on the first axis, the Laotian, Vietnamese, and Indonesian chicken breeds were clearly separated from the other breeds. In addition, WLG and MGN were completely isolated from the other breeds on the second axis, whereas the Asian chicken breeds were not clearly separated from each other on this axis. On the other hand, CON and KNO were separated from the other breeds on the third axis.

In conclusion, the genetic diversity of the KNC and imported breeds was lower than that of the other Asian chicken breeds, whereas their genetic uniformity was higher, except in the MGN, KGPS, and LCH. Therefore, selection for the specific characteristics of Asian chicken breeds used in this study is necessary to increase their genetic uniformity. Moreover, estimating the genetic diversity between the 22 Asian chicken breeds is the first step in a strategic plan for the genetic characterization and conservation of these breeds. Although the sample size of some of the breeds was small, our findings are meaningful in that the study was conducted using various breeds from different countries. Additionally, this study may be useful as an initial guide for defining conservation objectives, for designing future investigations of genetic variation, and for developing conservation strategies for 22 Asian chicken breeds. However, further research is required to elucidate the specific reasons for the genetic differences between the Asian breeds and the native Korean and imported breeds.

## Figures and Tables

**Table 1:** Description of the 22 chicken breeds used in this study

Country	Abbr.	Breed	No. of samples	Source
Korea	RIR	Rhode Island Red	32	NIAS
	WLG	White Leghorn	32	
	CON	Cornish	32	
	KNG	Korean Grayish- brown	32	
	KNB	Koran Black	32	
	KNR	Korean Reddish-brown	32	
	KNW	Korean White	32	
	KNY	Korean Yellowish-brown	32	
	KNO	Korean Ogye	32	
Mongolia	MGN	Mongolian Nuthiin bor	9	NCLG
Indonesia	INK	Indonesian KUB	7	IRIAP
	INS	Indonesian Sensi	8	
	ING	Indonesian Gaok	8	
Kyrgyzstan	KGPS	Kyrgyzstani GPS-H	24	NASK
Laos	LYO	Laotian York	7	LRC
	LCH	Laotian Chae	7	
	LBB	Laotian Black bone	11	
	LOU	Laotian Ou	10	
Nepal	NPS	Nepalese Sakini	27	NARC
Sri Lanka	SBC	Sri Lankan Junglefowl	15	VRI
Vietnam	VTN	Vietnamese Noi	25	IASVN
	VNH	Vietnamese Ninh Hoa	23	

NIAS, National Institute of Animal Science; NCLG, The National Centre for Livestock Genebank; IRIAP, Indonesia Research Institute for Animal Production; NASK, National Academy of Sciences of the Kyrgyz Republic; LRC, Livestock Research Center; NARC, Nepal Agricultural Research Council; VRI, Veterinary Research Institute; IASVN, Institute of Animal Sciences for Sourthern Vietnam.

**Table 2:** The statistical analysis of heterozygosity and polymorphism information content using 20 microsatellite markers

Locus	NA	*H*_exp_	*H*_obs_	PIC
LEI0192	20	0.661±0.040	0.536±0.049	0.596±0.039
MCW0233	5	0.555±0.048	0.593±0.060	0.476±0.043
MCW0078	6	0.400±0.039	0.454±0.054	0.327±0.032
ADL0278	8	0.610±0.029	0.667±0.048	0.534±0.027
MCW0193	10	0.696±0.029	0.735±0.034	0.624±0.030
MCW0240	14	0.707±0.035	0.650±0.039	0.640±0.037
LEI0094	19	0.672±0.030	0.668±0.039	0.601±0.030
MCW0295	11	0.646±0.036	0.638±0.043	0.577±0.033
MCW0145	11	0.644±0.036	0.591±0.041	0.576±0.034
MCW0330	9	0.616±0.037	0.639±0.045	0.535±0.035
LEI0099	9	0.577±0.032	0.573±0.045	0.491±0.032
MCW0252	11	0.677±0.029	0.689±0.036	0.602±0.030
LEI0135	8	0.615±0.041	0.599±0.044	0.541±0.039
ADL0176	11	0.714±0.021	0.622±0.028	0.640±0.022
MCW0322	4	0.479±0.036	0.440±0.041	0.392±0.030
MCW0016	9	0.613±0.048	0.619±0.056	0.545±0.044
LEI0096	14	0.629±0.038	0.634±0.043	0.562±0.036
MCW0103	3	0.348±0.036	0.382±0.046	0.272±0.027
MCW0037	4	0.598±0.025	0.562±0.035	0.507±0.024
LEI0166	7	0.510±0.040	0.526±0.055	0.424±0.035
Mean	9.65	0.598±0.022	0.591±0.020	0.523±0.023

NA, number of alleles; *H*_exp_, expected heterozygosity; *H*_obs_, observed heterozygosity; PIC, polymorphism information content.

**Table 3:** Genetic diversity parameters in 22 chicken breeds

Population	Sample size	No alleles	*H*_exp_	*H*_obs_	PIC
RIR	32	2.80±1.01	0.461±0.042	0.448±0.020	0.391±0.038
WLG	32	2.90±1.25	0.372±0.055	0.384±0.019	0.325±0.049
CON	32	3.40±1.05	0.534±0.032	0.528±0.020	0.461±0.031
KNG	32	3.00±1.08	0.426±0.040	0.466±0.020	0.364±0.034
KNB	32	4.00±1.86	0.569±0.033	0.583±0.020	0.503±0.033
KNR	32	4.35±1.42	0.611±0.038	0.617±0.019	0.550±0.037
KNW	32	3.85±1.42	0.578±0.029	0.566±0.020	0.508±0.031
KNY	32	4.35±1.53	0.586±0.037	0.581±0.020	0.530±0.037
KNO	32	3.20±1.06	0.513±0.043	0.509±0.020	0.443±0.038
MGN	9	2.15±0.93	0.382±0.058	0.515±0.037	0.306±0.048
INK	7	4.45±1.61	0.717±0.036	0.664±0.040	0.614±0.037
INS	8	3.90±1.29	0.654±0.036	0.631±0.038	0.560±0.035
ING	8	4.30±1.34	0.696±0.030	0.656±0.038	0.599±0.031
KGPS	24	4.25±1.16	0.635±0.023	0.618±0.022	0.563±0.024
LYO	7	4.10±1.41	0.654±0.041	0.679±0.039	0.557±0.039
LCH	7	4.25±1.21	0.684±0.035	0.690±0.039	0.584±0.033
LBB	11	4.05±1.50	0.637±0.032	0.686±0.031	0.551±0.034
LOU	10	4.40±1.96	0.683±0.030	0.619±0.035	0.585±0.034
NPS	27	6.50±3.02	0.718±0.027	0.666±0.020	0.663±0.030
SBC	15	4.50±1.24	0.654±0.023	0.563±0.029	0.580±0.023
VTN	25	5.85±2.11	0.686±0.027	0.640±0.021	0.624±0.031
VNH	23	6.30±2.36	0.709±0.026	0.687±0.022	0.650±0.030

*H*_exp_, expected heterozygosity; *H*_obs_, observed heterozygosity; PIC, polymorphism information content; RIR, Rhode Island Red; WLG, White Leghorn; CON, Cornish; KNG, Korean Grayish-brown; KNB, Korean Black; KNR, Korean Reddish-brown; KNW, Korean White; KNY, Korean Yellowish-brown; KNO, Korean Ogye; MGN, Mongolian Nuthiin bor; INK, Indonesian KUB; INS, Indonesian Sensi; ING, Indonesian Gaok; KGPS, Kyrzyzstani GPS-H; LYO, Laotian York; LCH, Lotian Chae; LBB, Laotian Black Bone; LOU, Latotian Ou; NPS, Nepalese Sakini; SBC, Sri Lankan Junglefowl; VTN, Vietnamese Ninh Hoa; VNH, Vietnamese Ninh Hoa.

## References

[1] FAO Global plan of action for animal genetic resources and the Interlaken declaration.

[2] (c2015). About the Nagoya Protocol [internet]. Convention on biological diversity.

[3] Ajmone-Marsan P, Garcia JF, Lenstra JA (2010). On the origin of cattle: how aurochs became cattle and colonized the world. Evol Anthropol.

[4] Felius M, Theunissen B, Lenstra JA (2015). Conservation of cattle genetic resources: the role of breeds. J Agric Sci.

[5] Scherf BD, Pilling D (2015). The second report on the state of the world's animal genetic resources for food and agriculture.

[6] Biscarini F, Nicolazzi EL, Stella A, Boettcher PJ, Gandini G (2015). Challenges and opportunities in genetic improvement of local livestock breeds. Front Genet.

[7] Rischkowsky B, Pilling D (2007). The state of the world's animal genetic resources for food and agriculture.

[8] Kaya M, Yildiz MA (2008). Genetic diversity among Turkish native chickens, Denizli and Gerze, estimated by microsatellite markers. Biochem Genet.

[9] Medugorac I, Medugorac A, Russ I (2009). Genetic diversity of European cattle breeds highlights the conservation value of traditional unselected breeds with high effective population size. Mol Ecol.

[10] Groeneveld LF, Lenstra JA, Eding H (2010). Genetic diversity in farm animals–a review. Anim Genet.

[11] Lenstra JA, Groeneveld LF, Eding H (2012). Molecular tools and analytical approaches for the characterization of farm animal genetic diversity. Anim Genet.

[12] Dawson DA, Ball AD, Spurgin LG (2013). High-utility conserved avian microsatellite markers enable parentage and population studies across a wide range of species. BMC Genomics.

[13] Bowcock AM, Ruiz-Linares A, Tomfohrde J, Minch E, Kidd JR, Cavalli-Sforza LL (1994). High resolution of human evolutionary trees with polymorphic microsatellites. Nature.

[14] Cheng HH, Crittenden LB (1994). Microsatellite markers for genetic mapping in the chicken. Poult Sci.

[15] Hillel J, Groenen MAM, Tixier-Boichard M (2003). Biodiversity of 52 chicken populations assessed by microsatellite typing of DNA pools. Genet Sel Evol.

[16] Abdul-Muneer PM (2014). Application of microsatellite markers in conservation genetics and fisheries management: recent advances in population structure analysis and conservation strategies. Genet Res Int.

[17] Bakoumé C, Wickneswari R, Siju S, Rajanaidu N, Kushairi A, Billotte N (2015). Genetic diversity of the world’s largest oil palm (*Elaeis guineensis* Jacq.) field genebank accessions using microsatellite markers. Genet Resour Crop Evol.

[18] El-Esawi MA, Germaine K, Bourke P, Malone R (2016). Genetic diversity and population structure of *Brassica oleracea* germplasm in Ireland using SSR markers. C R Biol.

[19] Cuc NTK, Simianer H, Eding H (2010). Assessing genetic diversity of Vietnamese local chicken breeds using microsatellites. Anim Genet.

[20] Dorji N, Duangjinda M, Phasuk Y (2012). Genetic characterization of Bhutanese native chickens based on an analysis of Red Junglefowl (*Gallus gallus gallus* and *Gallus gallus spadecieus*), domestic Southeast Asian and commercial chicken lines (*Gallus gallus domesticus*). Genet Mol Biol.

[21] Seo JH, Lee JH, Kong HS (2017). Assessment of genetic diversity and phylogenetic relationships of Korean native chicken breeds using microsatellite markers. Asian-Australas J Anim Sci.

[22] Fathi M, El-Zarei M, Al-Homidan I, Abou-Emera O (2018). Genetic diversity of Saudi native chicken breeds segregating for naked neck and frizzle genes using microsatellite markers. Asian-Australas J Anim Sci.

[23] Park S (2000). Microsatellite Toolkit for MS Excel 97 or 2000.

[24] Ota T (1993). DISPAN: genetic distance and phylogenetic analysis.

[25] Felsenstein J (1993). PHYLIP (phylogeny inference package), version 3.5 c: Joseph Felsenstein.

[26] Page RDM (1996). Tree View: An application to display phylogenetic trees on personal computers. Bioinformatics.

[27] Pritchard JK, Stephens M, Donnelly P (2000). Inference of population structure using multilocus genotype data. Genetics.

[28] Earl DA, vonHoldt BM (2012). STRUCTURE HARVESTER: a website and program for visualizing STRUCTURE output and implementing the Evanno method. Conserv Genet Resour.

[29] Evanno G, Regnaut S, Goudet J (2005). Detecting the number of clusters of individuals using the software STRUCTURE: a simulation study. Mol Ecol.

[30] Jakobsson M, Rosenberg NA (2007). CLUMPP: a cluster matching and permutation program for dealing with label switching and multimodality in analysis of population structure. Bioinformatics.

[31] Rosenberg NA (2004). DISTRUCT: a program for the graphical display of population structure. Mol Ecol Notes.

[32] Jombart T (2008). adegenet: a R package for the multivariate analysis of genetic markers. Bioinformatics.

[33] Allaire J (2012). RStudio: integrated development environment for R.

[34] Zanetti E, De Marchi M, Dalvit C, Cassandro M (2010). Genetic characterization of local Italian breeds of chickens undergoing *in situ* conservation. Poult Sci.

[35] Suh S, Sharma A, Lee S (2014). Genetic diversity and relationships of Korean chicken breeds based on 30 microsatellite markers. Asian-Australas J Anim Sci.

[36] Mahammi FZ, Gaouar SBS, Laloë D (2016). A molecular analysis of the patterns of genetic diversity in local chickens from western Algeria in comparison with commercial lines and wild jungle fowls. J Anim Breed Genet.

[37] Botstein D, White RL, Skolnick M, Davis RW (1980). Construction of a genetic linkage map in man using restriction fragment length polymorphisms. Am J Hum Genet.

[38] Seo SW (2014). Molecular genetic evaluation of Korean domestic animal genetic resources using microsatellite markers [dissertation].

[39] Leroy G, Verrier E, Meriaux JC, Rognon X (2009). Genetic diversity of dog breeds: between-breed diversity, breed assignation and conservation approaches. Anim Genet.

