# Supplementary material for: Estimating genetic diversity and population structure of 22 chicken breeds in Asia using microsatellite markers
Source: Asian-Australas J Anim Sci. 2020 Apr 13;33(12):1896–904. doi: 10.5713/ajas.19.0958 (PMC7649407; doi:10.5713/ajas.19.0958)
Supplement: Supplementary file 1 [file ajas-19-0958-suppl.pdf]

Supplemetntary Table A. Information of 20 microsatellite markers which used in this study

Locus	Dye	Chromosome	Allele range (bp)	Primer sequence (5`-3`) Forward / Reverse
ADL0176	VIC	2	168 – 208	TTG TGG ATT CTT GGT GGT AGC<br>TTC TCC CGT AAC ACT CGT CA
LEI0096		2	216 – 250	GAT CAG ATT GGT TCC CTG TG<br>TGG GTG AAG TTT CCT CGT AG
LEI0135		28	122 – 154	CAC AAT GAA GGA TGA ATA GTG C<br>AAT TCA CAG TTA CAC CTG AGG
MCW0078		5	129 - 159	CCA CAC GGA GAG GAG AAG GTC T<br>TAG CAT ATG AGT GTA CTG AGC TTC
MCW0193		5	295 - 319	TAT TCA ATA GAG TTA CGC TGT C<br>ATT ACG TCT GCA CCA GTA CAG
MCW0240		4	165 – 195	CCA AAC CGG TGT CAC CTA CTG<br>GGT TAT TTC TTC AGT GAC TTC C
MCW0103		3	269 - 273	AAC TGC GTT GAG AGT GAA TGC<br>TTT CCT AAC TGG ATG CTT CTG
MCW0330		17	256 – 300	TGG ACC TCA TCA GTC TGA CAG<br>AAT GTT CTC ATA GAG TTC CTG C
ADL0278	NED	8	109 – 123	CCA GCA GTC TAC CTT CCT AT<br>TGT CAT CCA AGA ACA GTG TG
LEI0094		1	245 – 285	GAT CTC ACC AGT ATG AGC TGC<br>TCT CAC ACT GTA ACA CAG TGC
LEI0166		3	340 – 356	CTC CTG CCC TTA GCT ACG CA<br>TAT CCC CTG GCT GGG AGT TT
LEI0192		6	244 – 370	TGC CAG AGC TTC AGT CTG T<br>GTC ATT ACT GTT ATG TTT ATT GC
MCW0233		27	202 - 218	TCC AGC AGT AAG TAT AGC TGC<br>TGT TAG CTG CAG GGT ATT AGC
MCW0252		3	287 – 303	CTG CTC AAG CCC ATC GAC TTC C<br>CGA TAA CAT CTG ACA CTG CC
MCW0295		4	76 - 108	ATC ACT ACA GAA CAC CCT CTC<br>ATG TAT GCA CGC AGA TAT CC
MCW0322		13	238 - 258	GAT CTC CCT AGC TAC AAA CC<br>CTT CCG CCT TCT TGA GAG TC
LEI0099	PET	12	111 – 133	GAT CTG GCA GAA CAG AAA CAG<br>ATA TTT CAC ACC TGA CCT GCG
MCW0016		3	134 – 163	ATG GCG CAG AAG GCA AAG CGA TAT<br>TGG CTT CTG AAG CAG TTG CTA TGG
MCW0145		1	180 - 220	ACT TTA TTC TCC AAA TTT GGC T<br>AAA CAC AAT GGC AAC GGA AAC
MCW0037	6-FAM	3	152 - 158	ACC GGT GCC ATG AAT TAC CTA TTA<br>GAA AGC TAC CAT GAC ACT GCG AAA

Supplemetntary Table B. Matrix of D_A genetic distances observed among 22 chicken breeds

	RIR	WLG	CON	KNG	KNB	KNR	KNW	KNY	KNO	MGN	INK	INS	ING	KGPS	LYO	LCH	LBB	LOU	NPS	SBC	VTN	VNH
RIR	0.000																					
WLG	0.455	0.000																				
CON	0.282	0.393	0.000																			
KNG	0.279	0.445	0.416	0.000																		
KNB	0.247	0.359	0.289	0.284	0.000																	
KNR	0.280	0.370	0.276	0.269	0.181	0.000																
KNW	0.300	0.341	0.336	0.265	0.235	0.244	0.000															
KNY	0.317	0.422	0.294	0.236	0.235	0.178	0.235	0.000														
KNO	0.381	0.373	0.287	0.356	0.254	0.246	0.384	0.314	0.000													
MGN	0.457	0.175	0.444	0.503	0.439	0.455	0.462	0.458	0.441	0.000												
INK	0.360	0.413	0.355	0.351	0.324	0.266	0.328	0.283	0.333	0.449	0.000											
INS	0.457	0.405	0.447	0.368	0.426	0.313	0.355	0.346	0.405	0.443	0.221	0.000										
ING	0.389	0.432	0.379	0.388	0.334	0.285	0.337	0.344	0.423	0.486	0.244	0.279	0.000									
KGPS	0.306	0.328	0.243	0.336	0.277	0.224	0.325	0.260	0.330	0.373	0.355	0.408	0.371	0.000								
LYO	0.378	0.445	0.414	0.336	0.358	0.315	0.389	0.309	0.366	0.448	0.210	0.230	0.294	0.334	0.000							
LCH	0.349	0.497	0.353	0.399	0.347	0.332	0.387	0.289	0.402	0.483	0.313	0.343	0.309	0.370	0.266	0.000						
LBB	0.427	0.371	0.421	0.427	0.420	0.349	0.375	0.363	0.413	0.410	0.267	0.305	0.265	0.387	0.302	0.326	0.000					
LOU	0.323	0.414	0.386	0.285	0.310	0.272	0.309	0.288	0.356	0.479	0.198	0.237	0.258	0.302	0.154	0.276	0.252	0.000				
NPS	0.237	0.292	0.219	0.292	0.195	0.172	0.248	0.215	0.231	0.332	0.193	0.265	0.257	0.171	0.223	0.228	0.241	0.179	0.000			
SBC	0.222	0.396	0.311	0.260	0.200	0.207	0.237	0.221	0.338	0.415	0.263	0.326	0.287	0.246	0.287	0.295	0.331	0.227	0.155	0.000		
VTN	0.313	0.365	0.308	0.322	0.255	0.198	0.306	0.266	0.294	0.421	0.157	0.231	0.232	0.257	0.208	0.242	0.215	0.152	0.131	0.203	0.000	
VNH	0.324	0.395	0.304	0.332	0.261	0.210	0.297	0.252	0.296	0.437	0.164	0.225	0.210	0.271	0.208	0.241	0.221	0.148	0.128	0.214	0.086	0.000

RIR, Rhode Island Red; WLG, White Leghorn; CON, Cornish; KNG, Korean Grayish-brown; KNB, Korean Black; KNR, Korean Reddish-brown; KNW, Korean White; KNY, Korean Yellowish-brown; KNO, Korean Ogye; MGN, Mongolian Nuthiin bor; INK, Indonesian KUB; INS, Indonesian Sensi; ING, Indonesian Gaok; KGPS, Kyrzyzstani GPS-H; LYO, Laotian York; LCH, Lotian Chae; LBB, Laotian Black Bone; LOU, Latotian Ou; NPS, Nepalese Sakini; SBC, Sri Lankan Junglefowl; VTN, Vietnamese Ninh Hoa; VNH, Vietnamese Ninh Hoa.

Supplemetntary Table C. Population of membership of each 22 chicken breeds genotypes with 20 microsatellite markers in the 20 inferred clusters using STRUCTURE analysis

POP	Cluster																				N
	1	2	3	4	5	6	7	8	9	10	11	12	13	14	15	16	17	18	19	20	
RIR	0.005	0.003	0.002	0.002	0.002	0.003	0.002	0.003	0.005	0.002	0.002	0.005	0.002	0.004	**0.945**	0.002	0.002	0.003	0.002	0.003	32
WLG	0.002	0.003	0.002	0.005	0.003	0.006	0.002	0.002	0.002	0.002	0.002	0.003	0.002	0.003	0.002	0.002	0.002	**0.951**	0.002	0.002	32
CON	0.004	0.003	0.004	0.003	0.003	0.004	0.01	0.003	**0.88**	0.004	0.002	0.004	0.002	0.004	0.051	0.003	0.003	0.005	0.004	0.003	32
KNG	0.003	0.002	0.003	0.003	0.003	0.05	0.002	0.003	0.049	0.002	0.002	0.003	0.002	**0.854**	0.006	0.003	0.002	0.002	0.003	0.003	32
KNB	0.006	0.009	0.006	0.004	0.003	0.005	0.006	0.01	0.005	0.014	0.004	**0.891**	0.005	0.007	0.007	0.004	0.003	0.004	0.003	0.004	32
KNR	0.014	0.006	0.019	0.006	0.003	0.006	0.008	**0.845**	0.006	0.007	0.005	0.014	0.006	0.007	0.014	0.007	0.004	0.013	0.006	0.005	32
KNW	0.006	0.003	0.007	0.004	0.003	**0.889**	0.003	0.005	0.007	0.007	0.003	0.009	0.004	0.008	0.009	0.007	0.003	0.016	0.003	0.003	32
KNY	0.01	0.007	**0.878**	0.005	0.004	0.007	0.01	0.01	0.011	0.006	0.003	0.008	0.004	0.008	0.008	0.004	0.004	0.006	0.003	0.005	32
KNO	0.003	0.003	0.003	0.003	0.003	0.003	0.003	0.005	0.004	**0.924**	0.002	0.01	0.005	0.005	0.003	0.003	0.002	0.013	0.002	0.002	32
MGN	0.005	0.004	0.003	0.004	0.004	0.002	0.003	0.002	0.002	0.003	0.003	0.003	0.002	0.05	0.005	0.006	0.003	**0.889**	0.004	0.005	9
INK	0.008	0.008	0.01	0.051	0.05	0.02	0.003	0.009	0.008	0.019	**0.472**	0.007	0.027	0.053	0.007	0.108	0.098	0.005	0.024	0.012	7
INS	0.005	0.005	0.006	0.009	0.083	0.014	0.003	0.008	0.003	0.005	0.02	0.003	0.006	0.008	0.003	0.008	**0.795**	0.006	0.006	0.005	8
ING	0.007	0.006	0.024	0.396	0.015	0.006	0.004	0.01	0.004	0.005	0.021	0.011	0.018	0.005	0.011	0.019	**0.407**	0.005	0.012	0.016	8
KGPS	0.012	0.018	0.009	0.005	0.003	0.006	**0.863**	0.007	0.01	0.005	0.005	0.006	0.005	0.01	0.01	0.005	0.003	0.005	0.004	0.007	24
LYO	0.007	0.005	0.01	0.011	**0.634**	0.004	0.004	0.006	0.004	0.012	0.013	0.005	0.126	0.007	0.004	0.008	0.034	0.003	0.005	0.101	7
LCH	0.006	0.007	0.008	0.008	0.012	0.005	0.003	0.005	0.006	0.009	0.008	0.023	0.002	0.007	0.006	0.009	0.01	0.003	0.014	**0.849**	7
LBB	0.003	0.005	0.003	0.084	0.006	0.003	0.002	0.002	0.002	0.002	0.007	0.002	0.003	0.003	0.003	0.093	0.088	0.006	**0.676**	0.006	11
LOU	0.02	0.005	0.017	0.043	0.248	0.009	0.009	0.012	0.005	0.013	0.038	0.011	**0.253**	0.019	0.007	0.056	0.055	0.003	0.152	0.026	10
NPS	0.086	**0.297**	0.012	0.088	0.026	0.006	0.049	0.01	0.018	0.03	0.061	0.018	0.013	0.015	0.013	0.144	0.019	0.017	0.026	0.053	27
SBC	**0.683**	0.037	0.032	0.014	0.008	0.017	0.006	0.01	0.011	0.006	0.014	0.026	0.006	0.035	0.022	0.034	0.009	0.006	0.007	0.018	15
VTN	0.017	0.015	0.005	0.05	0.009	0.004	0.006	0.01	0.006	0.006	**0.521**	0.011	0.168	0.014	0.006	0.076	0.017	0.009	0.028	0.021	25
VNH	0.008	0.028	0.011	0.041	0.018	0.006	0.012	0.013	0.014	0.015	**0.388**	0.016	0.067	0.012	0.006	0.267	0.038	0.005	0.018	0.018	23

RIR, Rhode Island Red; WLG, White Leghorn; CON, Cornish; KNG, Korean Grayish-brown; KNB, Korean Black; KNR, Korean Reddish-brown; KNW, Korean White; KNY, Korean Yellowish-brown; KNO, Korean Ogye; MGN, Mongolian Nuthiin bor; INK, Indonesian KUB; INS, Indonesian Sensi; ING, Indonesian Gaok; KGPS, Kyrzyzstani GPS-H; LYO, Laotian York; LCH, Lotian Chae; LBB, Laotian Black Bone; LOU, Latotian Ou; NPS, Nepalese Sakini; SBC, Sri Lankan Junglefowl.

Maximum numerial value for each breeds are in bold.
